# Evaluation of Cytotoxicity and Antifertility Effect of *Artemisia kopetdaghensis*

**DOI:** 10.1155/2014/745760

**Published:** 2014-03-10

**Authors:** Davood Oliaee, Mohammad Taher Boroushaki, Naiime Oliaee, Ahmad Ghorbani

**Affiliations:** ^1^Student Research Committee, Mashhad University of Medical Sciences, Mashhad 913750345, Iran; ^2^Pharmacological Research Center of Medicinal Plants, School of Medicine, Mashhad University of Medical Sciences, Mashhad 9177948564, Iran; ^3^Department of Pharmacology, School of Medicine, Mashhad University of Medical Sciences, Mashhad 9177948564, Iran

## Abstract

To date, there is no report on safety of *Artemisia Kopetdaghensis*. This study aimed to determine the possible undesirable effects of *A. Kopetdaghensis* on reproduction of female rats. The pregnant rats were treated (i.p.) with vehicle or 200 and 400 mg/kg of *A. Kopetdaghensis* hydroalcoholic extract from the 2nd to 8th day of pregnancy. Then, number and weight of neonates, duration of pregnancy, and percent of dead fetuses were determined. Also, cytotoxicity of this plant was tested using fibroblast (L929) and ovary (Cho) cell lines. The *A. Kopetdaghensis* had no significant effect on duration of pregnancy, average number of neonates, and weight of neonates. However, administration of 200 and 400 mg/kg of the extract led to 30 and 44% abortion in animals, respectively. The extract at concentrations ≥200 *μ*g/mL significantly (*P* < 0.001) inhibited the proliferation of L929 fibroblast cells. Regarding the Cho cells, the extract induced toxicity only at concentration of 800 *μ*g/mL (*P* < 0.01). Our results showed that continuous consumption of *A. Kopetdaghensis* in pregnancy may increase the risk of abortion and also may have toxic effect on some cells.

## 1. Introduction

Today, medicinal plants are widely used around the world as an alternative to pharmaceutical drugs. Although herbal products are considered to have fewer adverse effects compared with synthetic drugs, they are not completely free from side effects or toxicity [1]. Adverse effects of medicinal plants may result from contamination of herbs with toxic metals, adulteration with active synthetic compounds, improperly prepared herbal products, misidentification of herbal ingredients, and inherent toxicity of certain herbs [2]. Therefore, the potential side effects of any medicinal plant need to be determined before its clinical applications. Special care should be taken when a herbal product is used by pregnant women, children, and geriatrics. Unfortunately, unlike those synthetic drugs not recommended for use in pregnancy because of known unwanted effects, there are insufficient data about undesirable maternal and perinatal consequences of use of herbal agents.

*Artemisia kopetdaghensis*, aromatic shrubs belonging to the Asteraceae family, is traditionally used in Iran for its anti-inflammatory, antimicrobial, antifungal, and sedative activities [3, 4]. However, to date, there is no report on safety or toxicity of this plant. Only Ebrahimi and coworkers reported that methanolic extract and essential oil of* A. kopetdaghensis* exhibited tumor growth induction at some concentrations and cytotoxicity at other concentrations [5]. The aim of the present study was to determine the possible undesirable effects of* A. kopetdaghensis* on reproduction of female rats. Also, the possible cytotoxicity of this plant was assessed using fibroblast and ovary cells* in vitro*.

## 2. Materials and Methods

### 2.1. Chemicals and Reagents

High glucose Dulbecco's Modified Eagles Medium (DMEM) and fetal bovine serum were purchased from Gibco. Penicillin, streptomycin, and 3-(4,5-Dimethyl-2-thiazolyl)-2,5-Diphenyl-2H-tetrazolium bromide (MTT) were obtained from Sigma. Dimethyl sulfoxide (DMSO) was purchased from Fluka. Tween 80 was purchased from Merck.

### 2.2. Preparation of Plant Extract

The fresh* A. kopetdaghensis* was collected from Gonabad (Eastern area of Iran) and identified by the herbarium of Ferdowsi University of Mashhad, Iran (voucher specimen number: 35205). The aerial parts of plant were cleaned and grounded to fine powder with a blender. Then, macerated extract was prepared as described previously [6, 7], briefly by suspension of 200 g of the powder in 500 mL of 50% ethanol and incubation for 72 h at 37°C. The hydroalcoholic extract was then dried on a water bath and the yield dissolved in distilled water containing 1% Tween 80.

### 2.3. Animals

Male and female Wistar rats (200–250 g) and female mice (26–32 g) were obtained from Laboratory Animals Research Center, Mashhad University of Medical Sciences (Iran) and housed in a room with controlled lighting (12 h dark, 12 h light) and temperature (22 ± 2°C). The animals were given standard pellets diet and water* ad libitum*. All animal procedures were in accordance with ethical guidelines approved by the Animal Care Use Committee of Shiraz University of Medical Sciences (Iran).

### 2.4. Evaluation of* A. kopetdaghensis* Effect on Reproduction

Prior to the mating, the female rats were isolated for 30 days to rule out preexisting pregnancy. Then, they were caged overnight with a male rat of proven fertility in the ratio of 1 : 1. Rats exhibiting vaginal plug on the following morning were separated, and that day was considered as the first day of pregnancy. The pregnant rats were randomized into three groups: (1) control group receiving 1% Tween 80 as vehicle (*n* = 8), (2) experimental rats treated with 200 mg/kg of* A. kopetdaghensis* extract (*n* = 10), and (3) experimental rats receiving 400 mg/kg of the plant extract (*n* = 9). The extract was injected intraperitoneally from the 2nd to the 8th day of pregnancy (early period of organogenesis). The animals were kept individually in cages until parturition. Then, number and weight of neonates, duration of pregnancy, and percent of dead fetuses were determined.

### 2.5. Acute Toxicity Determination

Acute toxicity of* A. kopetdaghensis* extract was evaluated by the method of Akhila et al. [8], as described in our previously published work [9]. Five groups of two mice received vehicle (1% Tween 80) or 400, 800, 1600, and 3200 mg/kg of the plant extract intraperitoneally. The treated animals were monitored for 24 h and also one week for mortality. The lowest dose which led to death of animals and the highest dose which did not kill any mice were recorded.

### 2.6. Cytotoxicity Assessment

The L929 (mouse fibroblast) and Cho (Chinese hamster ovary) cells were seeded in 96-well plates and cultured for 24 h in DMEM supplemented with 10% FBS, penicillin (100 units/mL), and streptomycin (100 *μ*g/mL) at 37°C and 5% CO_2_. Then, the medium was changed to fresh one containing vehicle (1% DMSO) or 50–800 *μ*g/mL of* A. kopetdaghensis* extract. The cells were further incubated for 24 h at 37°C and 5% CO_2_. At the end of the treatment, the cell proliferation was measured using MTT assay as previously described [10–12]. The assay was carried out using 2 culture plates, 4 wells for each concentration (*n* = 8).

### 2.7. Statistical Analysis

The values were compared using the one-way analysis of variance (ANOVA) followed by Tukey's post hoc test for multiple comparisons. The *P* values less than 0.05 were considered to be statistically significant. All results are presented as mean ± SEM.

## 3. Results

### 3.1. Effect of* A. kopetdaghensis* on Reproduction

**Figure 1:**
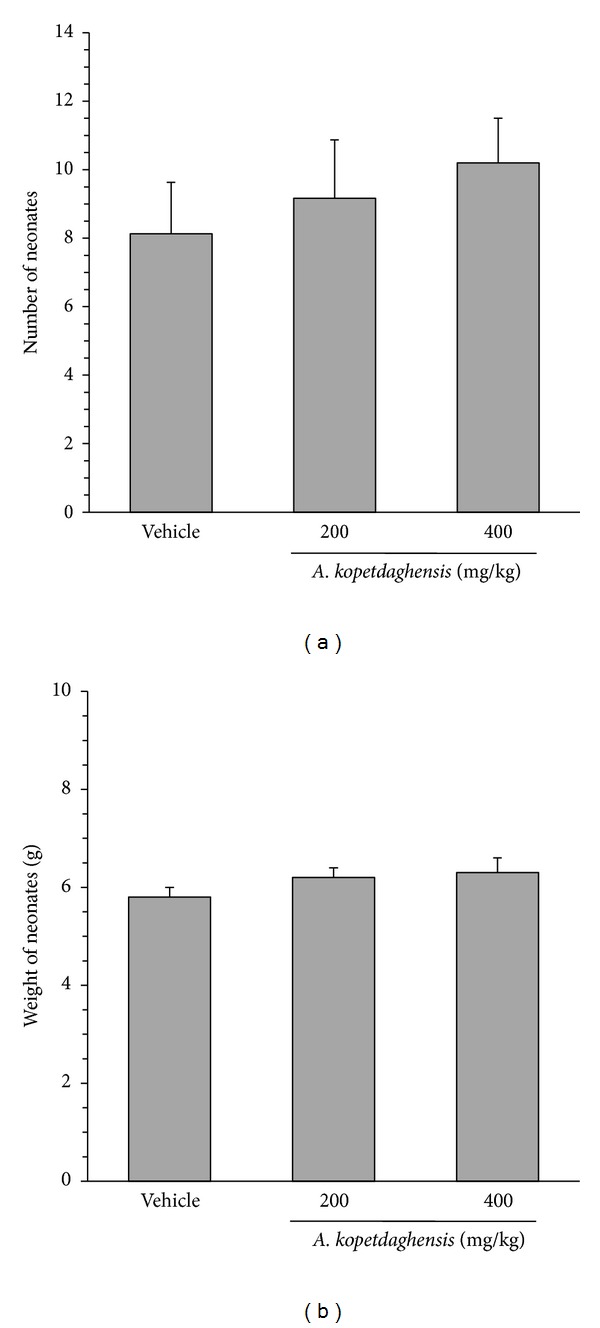
Effect of* Artemisia kopetdaghensis* hydroalcoholic extract on number (a) and weight (b) of neonates. The pregnant rats were treated (i.p.) with vehicle or 200 and 400 mg/kg of* A. kopetdaghensis* hydroalcoholic extract from the 2th to 8th day of pregnancy. Values are mean ± SEM (*n* = 8–10).

As shown in Table 1, the* A. kopetdaghensis* extract at concentrations of 200 and 400 mg/kg had no significant effect on duration of pregnancy. However, administration of 200 and 400 mg/kg of the extract led to 30 and 44% abortion in animals, respectively. The percent of dead neonates was 6% in control group and 7% and 0% in experimental groups treated with 200 and 400 mg/kg, respectively. The average number of the neonates in the animals receiving vehicle during pregnancy was 8.13 ± 1.5 (Figure 1(a)). None of the* A. kopetdaghensis* doses could cause a significant change in the neonate number. Likewise, the extract had virtually no significant effect on weight of neonates (Figure 1(b)).

### 3.2. Acute Toxicity of* A. kopetdaghensis*

Different groups of mice (*n* = 2) were treated with 400, 800, 1600, and 3200 mg/kg of* A. kopetdaghensis* hydroalcoholic extract. After 24 h, it was found that 1600 and 3200 mg/kg are the highest dose which did not kill any mice and the lowest dose which led to death of both mice, respectively. The treated animals were further monitored until one week and no mortality or any sign of toxicity was observed at doses ≤1600 mg/kg.

### 3.3. Cytotoxicity of* A. kopetdaghensis*

**Figure 2:**
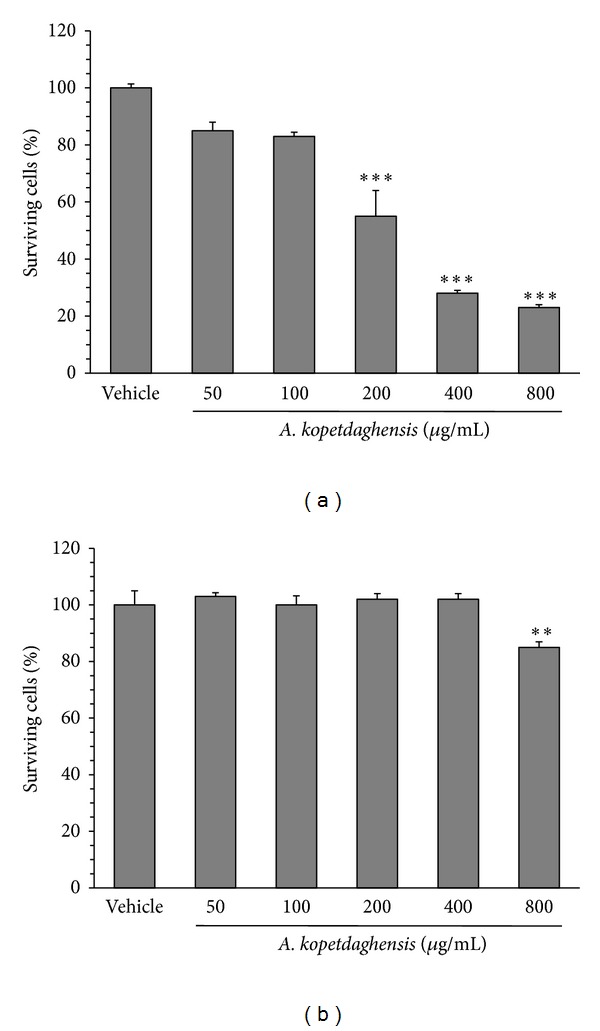
Effect of* Artemisia kopetdaghensis* hydroalcoholic extract on proliferation of fibroblast L929 (a) and Cho (b) cell lines. The cells were cultured for 24 h in the medium containing vehicle (1% DMSO) or 50–800 *μ*g/mL of* A. kopetdaghensis* extract. Values are mean ± SEM (*n* = 8).

Figure 2 shows the effect of* A. kopetdaghensis* hydroalcoholic extract on proliferation of L929 and Cho cells. Following incubation of L929 fibroblast cells with 50, 100, 200, 400, and 800 *μ*g/mL of the extract, approximately 15, 17, 45, 72, and 77% inhibition in cell growth, was observed, respectively, as compared with untreated cells. The cytotoxic effect of* A. kopetdaghensis* was statistically significant at concentrations ≥200 *μ*g/mL (*P* < 0.001). On the other hand, the extract induced toxicity on Cho cells only at concentration of 800 *μ*g/mL (*P* < 0.01). In the presence of 50, 100, 200, 400 and 800 *μ*g/mL of* A. kopetdaghensis*, surviving of Cho cells was 103 ± 1.3, 100 ± 3.2, 102 ± 2, 102 ± 2 and 85 ± 2%, respectively, as compared to untreated cells (100 ± 5).

## 4. Discussion

Many medicinal plants are used by pregnant women for their therapeutic effects. For example, it has been shown that about 36% of pregnant women in Norway use herbs [13]. However, these plants are consumed mostly based on personal experience or traditional knowledge and in most cases it is unclear how safe the use of them is during pregnancy. Previous studies highlighted that some of plants have different antifertility activities [14]. The present study was aimed to examine the possible toxic effects of* A. kopetdaghensis* on reproduction of female rats. Our data demonstrated that the plant extract has no effect on duration of pregnancy and number or weight of neonates. However, it can induce abortifacient effect when consumed at early period of pregnancy. This antifertility effect of* A. kopetdaghensis* has been also reported for some other plants of the Asteraceae family such as* Achillea millefolium* and* Aspilia africana* which showed antispermatogenic and antiovulatory activities, respectively [15, 16]. On the other hand, we observed that administration of* A. kopetdaghensis* extract (400 mg/kg) did not lead to stillbirth. The exact cause of this discrepancy should be explored in the future experiments. However, it may be attributed to high rate of abortion in animals receiving high concentration of the extract.

According to the previously published work, camphene, camphor, davanone, eucalyptol, eugenol, and geranial are of major components of* A. kopetdaghensis*. Camphor accounted for about 1.5 g/100 g of this plant [17]. Rabl and coworkers have reported that camphor crosses the placenta and may lead to abortion [18]. In another study, Linjawi reported that camphor induces significant structural changes on uterus of pregnant rats [19]. Therefore, it is rational to assume that camphor is involved in the abortifacient effect of* A. kopetdaghensis*.

Cytotoxicity evaluation of* A. kopetdaghensis* showed that its hydroalcoholic extract decreases proliferation of fibroblast cells. This finding may describe the camphor induced degeneration of luminal epithelium and decrease of endometrium thickness in uterus of pregnant animals [19].

In conclusion, our results showed that continuous consumption of* A. kopetdaghensis* in pregnancy may increase the risk of abortion and also may have toxic effect on some cells of body. Therefore, its continuous use is not recommended in pregnancy.

## Figures and Tables

**Table 1:** Effect of *Artemisia kopetdaghensis* on reproduction of female rats. The pregnant rats were treated (i.p.) with vehicle or *A. kopetdaghensis* hydroalcoholic extract from the 2th to 8th day of pregnancy. Values are mean ± SEM (*n* = 8–10).

Animal groups	Duration of pregnancy (day)	Abortion (%)	Percent of dead fetuses (%)
Control	22 ± 0.3	0	6
*A. kopetdaghensis* (200 mg/kg)	23 ± 0.3	30	7
*A. kopetdaghensis* (400 mg/kg)	22 ± 0.4	44	0
